# AGRN: accurate gene regulatory network inference using ensemble machine learning methods

**DOI:** 10.1093/bioadv/vbad032

**Published:** 2023-04-05

**Authors:** Duaa Mohammad Alawad, Ataur Katebi, Md Wasi Ul Kabir, Md Tamjidul Hoque

**Affiliations:** Department of Computer Science, University of New Orleans, New Orleans, LA 70148, USA; Department of Bioengineering, Northeastern University, Boston, MA 02115, USA; Center for Theoretical Biological Physics, Northeastern University, Boston, MA 02115, USA; Department of Computer Science, University of New Orleans, New Orleans, LA 70148, USA; Department of Computer Science, University of New Orleans, New Orleans, LA 70148, USA

## Abstract

**Motivation:**

Biological processes are regulated by underlying genes and their interactions that form gene regulatory networks (GRNs). Dysregulation of these GRNs can cause complex diseases such as cancer, Alzheimer’s and diabetes. Hence, accurate GRN inference is critical for elucidating gene function, allowing for the faster identification and prioritization of candidate genes for functional investigation. Several statistical and machine learning-based methods have been developed to infer GRNs based on biological and synthetic datasets. Here, we developed a method named AGRN that infers GRNs by employing an ensemble of machine learning algorithms.

**Results:**

From the idea that a single method may not perform well on all datasets, we calculate the gene importance scores using three machine learning methods—random forest, extra tree and support vector regressors. We calculate the importance scores from Shapley Additive Explanations, a recently published method to explain machine learning models. We have found that the importance scores from Shapley values perform better than the traditional importance scoring methods based on almost all the benchmark datasets. We have analyzed the performance of AGRN using the datasets from the DREAM4 and DREAM5 challenges for GRN inference. The proposed method, AGRN—an ensemble machine learning method with Shapley values, outperforms the existing methods both in the DREAM4 and DREAM5 datasets. With improved accuracy, we believe that AGRN inferred GRNs would enhance our mechanistic understanding of biological processes in health and disease.

**Availabilityand implementation:**

https://github.com/DuaaAlawad/AGRN.

**Supplementary information:**

Supplementary data are available at *Bioinformatics* online.

## 1 Introduction

Different cell types have their distinct gene expression profiles, and cells differentiate from one cell state to another by changing their expression profile via regulating gene transcription. In this regulatory mechanism, a transcription factor binds to the promoter of a gene target to modulate its expression. The causal interactions between the transcription factors and their target genes collectively can drive a biological process and are known as a gene regulatory network (GRN) (Marbach *et al.*, 2012). Hence, inferring accurate GRNs is essential for a mechanistic understanding of biological processes in healthy and pathological states (MacNeil, 2011). The availability of a massive collection of gene expression data allows for inference of high-throughput and large-scale network topology. Several computational methods for inferring GRNs from these expression data have been developed and employed in real-world applications (Liu Wei, 2020).

The inference of gene regulation considers an underdetermined problem because the number of possible interactions exceeds the number of measurements available. This underdetermined problem, which is viewed as a complex problem, has produced many algorithms that attempt various ways to address this inherent difficulty (Lim, 2013). To infer the topology of large GRNs, many researchers have made great efforts to solve the network inference problem. They presented different algorithms that often compute pair-wise information measures between genes (Chan, 2017). These algorithms can differ depending on the machine learning concept used to predict the regulation weights between gene pairs. Based on the level of supervision, there are three categories of machine learning methods: unsupervised, supervised and semi-supervised. Supervised learning is the process of giving a machine learning model labeled data. The labeled dataset typically comprises of data gained through experience, whereas unsupervised learning entails utilizing unlabeled data. In actuality, it is frequently impossible to secure labels in these circumstances. For example, there is insufficient data understanding, or the labeling is too expensive. While semi-supervised learning involves working with a dataset that is divided into two parts: a labeled component and an unlabeled half. This method is frequently employed when identifying the data or collecting labeled data is too difficult or costly. The labeled portion of the data may likewise be of poor quality.

Unsupervised methods infer GRNs from the expression data and include the following three types of methods: regression-based, information theory-based and correlation-based. In regression-based methods, target genes select transcription factors through sparse linear regression. Such a method is TIGRESS which uses the least angle regression feature selection technique paired with stability selection to tackle the network inference problem (Haury, 2012). Information theory-based methods tend to rank edges based on mutual information. ARACNE is a method that reconstructs GRNs based on a Gaussian kernel estimator to determine the mutual information between the expression profiles of genes with a sparsity constraint (Adam *et al.*, 2006). It filters out non-significant as well as indirect interactions. Correlation-based methods calculate correlations between gene pairs. Such a method is ANOVerence which proposed the eta-squared score (*η*^2^) as an alternate measure for evaluating gene dependencies (Robert Küffner, 2012). The method used analysis of variance to derive a non-parametric and non-linear correlation coefficient as gene importance scores. ANOVerence is fast and simple to use and does not require the input data to be discretized. In DREAM5, ANOVerence was rated the best performer on real-world expression data.

Supervised learning methods have been developed to train different classifiers that infer regulatory interactions. Many studies have demonstrated that carefully trained supervised models outperform unsupervised methods (Cerulo *et al.*, 2010; Maetschke Stefan, 2013; Mordelet and Vert, 2008). These supervised methods decompose the GRN inference problem into a large number of subproblems to estimate local models for characterizing the genes regulated by each transcription factor (Maetschke Stefan, 2013). A few such methods are GENIE3 (Huynh-Thu, 2010), PPCOR (Kim, 2015), LEAP (Specht and Li, 2017), PIDC (Chan *et al.*, 2017) and GRNBoost2 (Moerman *et al.*, 2019). Huynh-Thu *et al.* developed the GENIE3 algorithm, which used tree-based methods, random forest or extra tree regression to infer GRN (Huynh-Thu, 2010). An input gene’s importance in predicting a target gene’s expression pattern is interpreted as a possible regulatory link. The network is then reverse engineered by aggregating putative regulatory linkages across all genes to produce ratings of the interactions. The GENIE3 algorithm was the best performer in DREAM4 (Greenfield, 2010) and DREAM5 (Marbach *et al.*, 2012), two major GRN inference challenges held in 2009 and 2010. Furthermore, in PPCOR, Kim *et al.* computes the partial and semi-partial correlation coefficients for every pair of genes with respect to all the other variables to infer the gene regulatory network. In addition, LEAP, Alicia *et al.* reconstructs gene regulatory networks by calculating the Pearson correlation coefficient, while in PIDC, Chan *et al.* developed a fast, efficient algorithm that uses partial information decomposition (PID) to identify regulatory relationships between genes. Moreover, Moerman T *et al* introduced (GRNBoost2) which is a fast alternative for GENIE3, especially suited for datasets with tens of thousands of samples. Like GENIE3, GRNBoost2 trains a regression model to select the most important regulators for each gene in the dataset. GRNBoost2 achieves its efficiency by using stochastic Gradient Boosting Machine regression with early-stopping regularization to infer the network.

Semi-supervised learning methods also have been used to infer GRNs. For example, Patel and Wang (2015) presented semi-supervised approaches for GRN prediction based on random forests and support vector machines, two machine learning algorithms. Unlabeled data were used to train semi-supervised learning models. They investigated both inductive and transductive learning methods, using an iterative mechanism to generate reliable negative training data from the unlabeled data. They used gene expression data from *Escherichia coli* and *Saccharomyces cerevisiae* to evaluate the performance of their strategies using a semi-supervised algorithm.

With recent advances in deep learning, some methods predict gene regulatory relationships through a deep learning framework. Such a method is CNNC (Bar-Joseph, 2019), which employs a convolutional neural network (CNN) to predict GRN from single-cell RNA-seq expression data. The method transformed the expression data lacking locality into an image-like object that CNNs could operate well. Then CNNs were used to learn the gene interactions, causality inferences, functional assignments and disease gene predictions. In addition, Wang *et al.* proposed a gene regulatory graph neural network approach for reconstructing GRNs from scratch using gene expression data (Wang Juexin, 2020). They defined the GRN inference as a graph classification task, i.e. the algorithm determined whether a subgraph with two nodes at its center contained a link between them. A positive subgraph was formed by a linked pair of transcription factors and target gene together with their neighbors, whereas a negative subgraph was formed by an unlinked transcription factor and target gene pair together with their neighbors.

Although significant progress has been made, the GRN inference problem is far from being solved. In this study, we focus on improving the performance of the gene regulation prediction model using ensemble machine learning, which has emerged as a way to achieve better predictive performance than using single machine learning algorithms (Suraj *et al*., 2019; Zhang and Ma, 2012). Ensemble learning is a machine learning technique in which multiple models are trained to solve the same problem and then combined to produce better results (Dietterich, 2000). Ensemble methods aim to reduce individual machine learning models’ bias and/or variance by combining several of them into a robust (ensemble model) model that achieves better results. Additionally, this study uses the Shapley Additive Explanations (SHAP) as an importance score-based feature selection method (López de Prado, 2020) and consider as the first work that uses Shapley values as gene interactions scores. The SHAP value is one of the most widely used measures of feature importance by computing the contribution of each feature to the prediction. In this work, we explore several machine learning algorithms along with SHAP and propose a novel method named AGRN, which aims to find the importance scores for the links of the GRN from an ensemble machine learning algorithm. We combine the SHAP importance scores from three distinct methods, namely, extra tree regressor (ETR), random forest regressor (RFR) and support vector regressor (SVR). Furthermore, we optimize the hyperparameter of SVR and iteratively calculate importance scores using SVR by taking a subset from the dataset. Finally, we take the optimized weighted average of the scores to calculate the final importance score. Benchmarking results show that our ensemble-based method outperforms other comparable methods. We believe that the good performance of AGRN will be useful to predict GRNs more accurately, which can increase our understanding of how biological processes work in health and disease.

## 2 Methods

This section formally discusses the definition of the gene regulatory network (GRN) inference problem, the datasets we used to evaluate our method and the performance evaluation metrics. Finally, we discuss the AGRN framework to predict GRN.

### 2.1 Problem definition

The problem can be defined with expression data as a matrix where each row represents the expression levels of all G genes in one of the S samples.


$$\left[\begin{array}{ccc} x_{1,1} & x_{1,2} & \begin{array}{cc} \ldots & x_{1,G} \end{array}\\ x_{2,1} & x_{2,2} & \begin{array}{cc} \cdots & x_{2,G} \end{array}\\ \begin{array}{c} \vdots \\ x_{S,1} \end{array} & \begin{array}{c} \vdots \\ x_{S,2} \end{array} & \begin{array}{cc} \begin{array}{c} \ddots \\ \ldots \end{array} & \begin{array}{c} \vdots \\ x_{S,G} \end{array} \end{array} \end{array}\right],\;x_{s,g:\;\mathrm{the}\;\mathrm{expression}\;\mathrm{value}\;\mathrm{of}\;\mathrm{gene}\;g\;\mathrm{in}\;\mathrm{sample}\;s}$$

The algorithm’s output is then described as a directed graph in which each node represents a single gene, and a directed edge from node i to j indicates that gene i governs the expression of gene j (Joeri Ruyssinck, 2014). A score is assigned to each conceivable edge in the network, indicating the degree of certainty that this is a true regulatory link.

### 2.2 Datasets

This study uses gene expression data to infer the directed network topology of the target GRN. We do not make any further assumptions regarding whether the data was generated using gene knockouts, multifactorial perturbations, steady-state observations or other experimental settings. Also, self-regulatory interactions are ignored, and no time-related information is considered. Throughout this study, we used a directed topology setting similar to the setup used in the DREAM challenges (Alberto de la Fuente, 2014; Marbach *et al.*, 2009), allowing for a fair comparison of different techniques. The DREAM (elaborated as Dialogue for Reverse Engineering Assessments and Methods) challenges provide researchers with benchmark datasets for GRN inference to evaluate the findings. These challenges are considered to be the most comprehensive evaluations of GRN inference methods.

This research uses multifactorial perturbation data generated for the DREAM4 and DREAM5 (Marbach *et al.*, 2012) challenges. DREAM4 dataset comprises five synthetic networks, each with 100 genes, whereas the DREAM5 dataset comprises synthetic and real-world data from DREAM5. In this work, we use *in silico* data for the synthetic data and *E. coli* gene expression for the real-world data (Åkesson, *et al.*, 2021). Multifactorial expression data are static steady-state measurements obtained by slightly perturbing all genes simultaneously. Expression profiles obtained from biological replicates and different patients may be considered multifactorial data. These are easier and less expensive to obtain than knockout/knockdown or time-series data. As a result, multifactorial data are now more often used in practice. However, they are less useful for predicting edge directionality, making the task of inferring regulatory networks more difficult. Furthermore, the underlying network (the gold standard) is distributed along with the simulated expression data in the DREAM4 challenge to assess the quality of any inference made using the data, which is an important aspect. The numbers of genes and transcription factors (TFs) in each dataset used in this study are shown in (Supplementary Table S1**)**.

### 2.3 Performance evaluation metrics

AGRN ranks regulatory linkages from most confident to least confident. We employed a Precision-Recall (PR) curve (Jesse Davis, 2006) and a Receiver Operating Characteristic (ROC) curve (Yang Shengping, 2017) to evaluate the rankings. The PR curves give a more informative picture of an algorithm’s performance. For different thresholds on the significance scores, the PR curves show the relationship between the proportion of true positives among all predictions (precision) and the percentage of true positives that are retrieved (recall). In contrast, the ROC curve shows the true positive rate versus the false positive rate. We sorted the regulatory linkages by importance scores in descending order to evaluate the networks, keeping only the top 100 000 predictions similar to the setup used in the DREAM competition (Joeri Ruyssinck, 2014). The area under the ROC curve (AUROC) and the area under the PR curve (AUPR) are then calculated based on the benchmark data.

#### 2.3.1 Framework of AGRN

In AGRN, we determine the weight of the regulatory connection between the transcription factor (input gene) and the target gene, which is similar to how a machine learning approach determines feature importance. We adopt an ensemble machine learning approach to predicting an accurate GRN. The ensemble machine learning approach has recently been successfully applied to solve various bioinformatics problems (Joeri Ruyssinck, 2014). On a classification or regression task, a combination of machine learning models can harness the capabilities of a range of well-performing models and make predictions that outperform any single model in the ensemble (Dietterich, 2000). Although an algorithm may perform admirably on one problem, there is no reason to expect that it will perform equally well on another problem where the same assumptions may not hold. The ‘no free lunch’ (NFL) theorem (Adam, 2019) states that no single machine learning algorithm is universally the best-performing solution for all cases (Manisha Panta *et al.*, 2021; Sumaiya Iqbal, 2018).

To select the regressors for the ensemble, we examine the performance of seven individual regression algorithms, namely decision tree regressor (DTR), random forest regressor (RFR), extra tree regressor (ETR), extreme gradient boosting regressor (XGBR), Adaboost regressor (ABR), support vector regressor (SVR) and light gradient boosting machine (LGBM). The algorithms and their configuration details are briefly discussed here. DTR: It is a tree-based learning algorithm. A decision tree, consisting of decision nodes and leaf nodes, is incrementally developed by splitting the dataset into smaller subsets. The method can handle both categorical and numerical data (Sayed, 2012). RFR: It is a supervised learning algorithm that uses the ensemble learning method for regression (Breiman, 2001). It is a meta-estimator that aggregates many decision trees (bagging). The random forest creates trees in parallel, and these trees have no interaction. At the training time, the algorithm creates a large number of decision trees and outputs the average prediction (regression) of the individual trees. ETR: It is an ensemble machine learning method that uses averaging to improve predictive accuracy and control over-fitting by fitting a number of randomized decision trees from the original learning sample (Geurts, 2006). XGBR: This is another ML algorithm with the same principle of gradient boosting (Chen and Guestrin, 2016). The method uses more regularized model formalization to control over-fitting, which further leads to improved performance. XGBR also provides faster computational speed in addition to increased performance. ABR: This algorithm uses decision trees as weak learners added sequentially to the ensemble learning (Shrestha, 2004). The model’s predictions are employed in a future model to correct prediction mistakes. The method weighs the training dataset to focus on the training examples where previous models made prediction errors. SVR: This regression method allows us to determine how much error is acceptable in the model and choose a line or hyperplane that fits the data (Alawad *et al.*, 2020). We optimized the parameter epsilon regression and the cost parameter C using a Bayesian optimization algorithm. LGBM: This is a tree-based learning algorithm that grows the tree vertically and chooses the leaf based on the loss (Guolin Ke, 2017). The method uses the gradient boosting framework, which is a fast algorithm that can handle large datasets and has a shallow memory requirement.

Our proposed algorithm needs to quickly predict the GRN because identifying a network involving *p* genes requires rerunning the algorithm *p* times. The ensemble’s regressors are chosen so that each regressor’s underlying principle of learning is distinct from others, and the ensemble algorithm runs fast. By comparing the performance of the seven methods based on AUROC and AUPR (Supplementary Fig. S1), we found that RFR, ETR and SVR are the top-performing methods, where RFR and ETR are two tree-based regressors and SVR is a support vector machine-based regressor. So, we select these three methods to create our ensemble method AGRN.

RFR and ETR are two tree-based regressors that consist of many decision trees to improve the prediction performance. Each decision tree is constructed by recursively partitioning, which starts from the root node (known as the first parent); each node can be split into left and right child nodes. These nodes can then be further split and become parent nodes of their resulting children nodes. The split decision depends on the mean squared error (MSE), as shown in Equation 1 (James Bergstra, 2012; Sayed, 2012).


(1) $$\mathrm{MSE}\;(t)=\frac{1}{N_{t}}\sum_{i\in D_{t}}(y^{\left(i\right)}-\;\hat{y_{t}}{)}^{2}$$

(2) $$\hat{y_{t}}=\frac{1}{N_{t}}\sum_{i\in D_{t}}\;y^{\left(i\right)}$$

Here, $N_{t}\;$is the number of training samples at node t, $D_{t}$ is the training subset at node t, $y^{\left(i\right)}\;$is the true target value, and $\hat{y_{t}}$ is the predicted target value (sample mean).

Although RFR and ETR are similar in general concept (ensemble tree method), there are two main differences between them; RFR uses bootstrap replicas, which means it subsamples the input data with replacement. In contrast, ETR uses the whole original sample. Also, the selection of cut points to split the nodes is different; RFR chooses the optimum split (to reduce the variance), whereas ETR chooses it randomly.

In our ensemble-based method, GRN inference begins with determining the relevance of each feature in each regression problem. As a result, enhancing how we extract the feature’s importance scores is crucial, as it serves as a putative proof of a regulatory relationship between a gene pair. However, the conventional method for extracting feature importance from the tree-based method may be inconsistent, i.e. it is possible that the most important feature may not get the highest feature importance score (Lundberg, 2018). For example, the tree-based models can assign different scores to two equally important features depending on the level of splitting done with the features. The feature that splits first will be given a larger importance score. To overcome this limitation, we use the Shapley values as the feature importance scores in the two tree-based methods, RFR and ETR. Shapley Additive Explanations (SHAP) is a popular technique to explain machine learning models (Shapley, 1998). The idea behind SHAP is that the outcome of each possible combination (or coalition) of features should be considered when determining the importance of a single feature (Patel and Wang, 2015). Shapley values can be calculated using Equation 3, which represents an average over all possible subsets of marginal contribution for the features used in the model (Rozemberczki, *et al.*, 2022). where j is a feature, $\phi_{j}$ is the Shapley value for feature j, *p* is the number of features, S is the subset of features before adding the $j^{\mathrm{th}}\;$feature and ν is the prediction value.


(3) $$\phi_{j}\;(\nu )=\sum_{S\subseteq \{1,..,p)\setminus \{j\}}\frac{|S|!\;(p\;-\;|S|\;-\;1)!}{p!}\;(\nu (S\cup \;\{j\})\;-\;\nu \;(S))$$

We calculate the Shapley values for each gene of the sample, and they represent the input gene’s impact on the target genes that the input gene is related to.

On the other hand, we have selected SVR with a linear kernel because of its fast computation time. Several studies (Ganapathy and Peddinti, 2018; Huang *et al.*, 2018) have demonstrated the success of SVR and its efficient performance. SVR has characteristics that substantially impact our ensemble method AGRN, such as handling large feature spaces (Hua and Sun, 2001). Also, SVR has an excellent mathematical property that we can significantly improve the model’s performance by improving certain model parameters (Lee and Mangasarian, 2001). Suppose given training data {($x_{i},y_{i}$), i = 1, 2, …, n}, with input $x_{i},\;y_{i}\in R\;$and the main function for linear regression is (Smola and Schölkopf, 2004; Zheng, 2015): where w ∈R is the regression coefficient vector, and b ∈ R is the intercept. The SVR model employs a loss function, which is not sensitive if the difference between the observation ($y_{i}$) and the prediction ($w^{T}x_{i}\;+\;b$) is less than a predefined level E. We can obtain the SVR model from the linear model in Equation 4 by solving the following constrained minimization problem: where θ is the objective function of SVR, E is the predefined margin of error tolerance, $\xi_{i}\;={(\xi }_{1},\xi_{2},\ldots ,\xi_{n}{)}^{T}\;\mathrm{and}\;\xi_{i}^{*}\;=(\xi_{1}^{*},\xi_{2}^{*},\ldots ,\xi_{n}^{*}{)}^{T}$ where $\xi_{i}$, $\xi_{i}^{*}$ are the slack variables, which are part of the error that exceeds the error tolerance E. $\xi_{i}$ and $\xi_{i}^{*}$ can be considered as the effort we should make to bring the prediction ($w^{T}x_{i}\;+\;b$) to E-neighborhood of the observation $y_{i}$, if the distance between the prediction and observation $y_{i}$ is above the predefined error tolerance E. Also, $\frac{1}{2}\;w^{T}w$ is the term used to measure the regression model’s complexity, and the regularization parameter C > 0 balances the model complexity and the error on the training set made by the model. By using Lagrange Multiplier Method & Karush-Kuhn-Tucker (KKT) conditions, the previous optimization can be solved through its dual problem to obtain the following linear SVR in Equation (Zheng, 2015): where b represents the bias term, which can be calculated from the set of support vectors, and the coefficient terms $\sum_{i=1}^{n}(\alpha_{i}-\alpha_{i}^{*})\;x_{i}^{T}$ represent $w^{T}$ which is mentioned in Equation 4. This SVR model is implemented in the machine learning package Scikit-learn (Pedregosa, 2011).


(4) $$y=w^{T}x\;+\;b$$

(5) $$\begin{array}{l} \min_{w,\xi^{*},\;\xi }\;\theta (w,\xi^{*},\xi )\;=\;\frac{1}{2}\;w^{T}w\;+\;C[\sum_{i=1\;}^{n}\xi_{i}+\sum_{i=1}^{n}\xi_{i}^{*}]\\ \begin{array}{c} \mathrm{Subject}\;\mathrm{to}\;y_{i\;}-\;w^{T}x_{i\;}-\;b\;\leq E\;+\xi_{i}^{*}\\ \begin{array}{c} w^{T}x_{i\;}+\;b\;-\;y_{i\;}\;\leq E\;+\xi_{i}\\ \xi_{i}\;\geq \;0\;\xi_{i}^{*}\;\geq \;0\;\mathrm{for}\;i=1,\;2,\;\ldots ,\;n \end{array} \end{array} \end{array}$$

(6) $$y=\sum_{i=1}^{n}(\alpha_{i}-\alpha_{i}^{*})\;x_{i}^{T}\;x\;+\;b$$

To compute the feature importance for linear SVR, we calculate the coefficient score, which considers an efficient way to measure the feature importance in linear models of the machine learning algorithm. In addition, we found that calculating SHAP for SVR had no effect on performance and is computationally expensive. Moreover, in NIMEFI, the author found that sampling the dataset iteratively to calculate the importance scores from multiple SVR models produces better results than using the entire dataset with one single model (Joeri Ruyssinck, 2014). This work motivated us to choose SVR as one of the methods for our ensemble method. We follow the same setup that is used in NIMEFI (Joeri Ruyssinck, 2014). Using an expression data matrix consisting of rows and columns, the rows represent the observations while the columns represent the genes. In each iteration, $y_{i}$ represents a target gene while $y_{-i}$ represent as transcription factors (input genes). We select r subsampling of observation where r is a uniformly randomly generated integer number between 20% and 80% of the observations (rows of the expression data matrix). The subsampling process is repeated 200 times to generate 200 models for each target gene. We select the top five interactions in each iteration based on the importance score (coefficient score). Then we sum the importance scores from 200 iterations and use them as the final importance scores for SVR.

Setting SVR parameters is crucial since incorrect parameter selection can considerably impact accuracy. To optimize the SVR parameters, there are three common approaches, namely grid search (Yuting Sun, 2021), random search (James Bergstra, 2012; Ruder, 2017) and the probabilistic model-based approach (Jingqing Liu, 2006). In this work, we have used the Bayesian optimization algorithm, which is a probabilistic model-based approach for finding optimal hyperparameters (Feurer, *et al.*, 2014). The algorithm is better than the random search and faster than the grid search (James Bergstra, 2012). In SVR, we optimize two important parameters, the margin of tolerance (ϵ), within which no penalty is given to errors, and the regularization parameter (C), which means how much we want to avoid misclassification in each training data, as shown in Equation 5. (Pedregosa, 2011; Smola and Schölkopf, 2004; Wang Yisen, 2019).

After collecting the importance scores from the three methods, we take a weighted average to calculate the aggregate importance scores, as shown in Equation 7. where $R_{g}\;$is the final importance score for gene g, $\omega_{1}$is equal to 1, $\phi_{g}(R)$ is the Shapley value of gene g using RFR, $\omega_{2}$ is equal to 0.5, $\phi_{g}(E)$ is the Shapley value of gene g using ETR, $\omega_{3}\;$is equal to 0.1, $C_{g}(\mathrm{SV})$ is the coefficient value of gene g using SVR. We applied a grid search technique (Alawad *et al.*, 2020) to find the optimal weights to calculate the final importance scores. Finally, we calculated the z-score of final importance scores for genes in order to have a better understanding of the distribution of importance scores as shown in Equation 8. where $R_{g}\;$is the final importance score for gene g, μ is the mean of final importance scores, σ is the standard deviation of final importance scores for genes.


(7) $$R_{g}=\frac{\omega_{1}*\;(\phi_{g}(R))\;+\omega_{2}*\;(\phi_{g}(E))+\;\omega_{3}*\;(C_{g}(\mathrm{SV}))}{\omega_{1}+\omega_{2}+\omega_{3}}$$

(8) $$z-\mathrm{score}=\frac{R_{g}-\;\mu }{\sigma }$$

**Fig. 1.**
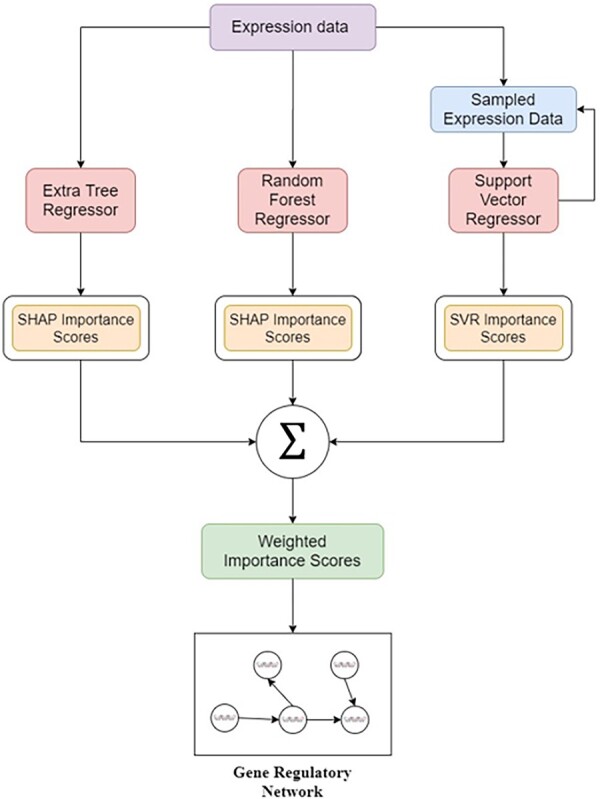
The framework of the AGRN to predict the gene regulatory network. AGRN combines three ML methods (ETR, RFR and SVR) and calculates the final importance scores using an optimized weighted average

The overall framework of AGRN to predict a GRN from the expression data is shown in Figure 1.

## 3 Results

In this section, first, we present the results of using seven machine learning algorithms to infer gene regulatory networks (GRN). Next, we demonstrate the performance of SHAP-based importance scores compared to the traditional importance scores. Finally, the proposed method, AGRN, is compared with other comparable methods in the literature.

The performance of the seven regression approaches is compared in terms of AUROC and AUPR (Supplementary Fig. S1). The results indicate that the best-performing model is SVR based on the average AUROC from the five networks, while the best performer is ETR based on the average AUPR (Supplementary Fig. S1). Results from the tree-based methods, ETR and RFR, are very close. So, we select the three best-performing methods: ETR, RFR and SVR, for further analysis.

After selecting the best three regression methods, we created an experimental setup with different combinations of SVR, ETR, RFR, ShapBasedOnRFR and ShapBasedOnETR, as shown in Supplementary Figures S2–S5. The ROC curve and the Precision-Recall curve are also shown in Supplementary Figures S6–S8. From these empirical results, we found that ShapBasedOnRFR, ShapBasedOnETR and SVR perform better in most of the DREAM4 and DREAM5 datasets. These motivated us to take the average importance scores from these three methods. However, we found that SVR performs well in some datasets but not in others. So, we took the weighted average of these three methods. We run a grid search algorithm in Network#1 of the DREAM4 dataset to find the optimal weights. Then, we use the optimal weights in the remaining datasets to compute the final importance score for AGRN. The selected optimal weights for RFR, ETR and SVR are 10, 5 and 1, respectively. In the following sections, we compare the performance of AGRN with the other comparable methods such as: GENIE3, GRNBoost2, PPCOR, LEAP and PIDC.

### 3.1 Comparison with some existing methods

To evaluate the effectiveness of AGRN, we have rerun five benchmarking methods that are mentioned in (Pratapa, *et al.*, 2020), such as GRNBoost2, PPCOR, PIDC, GEINE3 and LEAP, using four datasets from DREAM4 and three datasets from DREAM5.

**Fig. 2.**
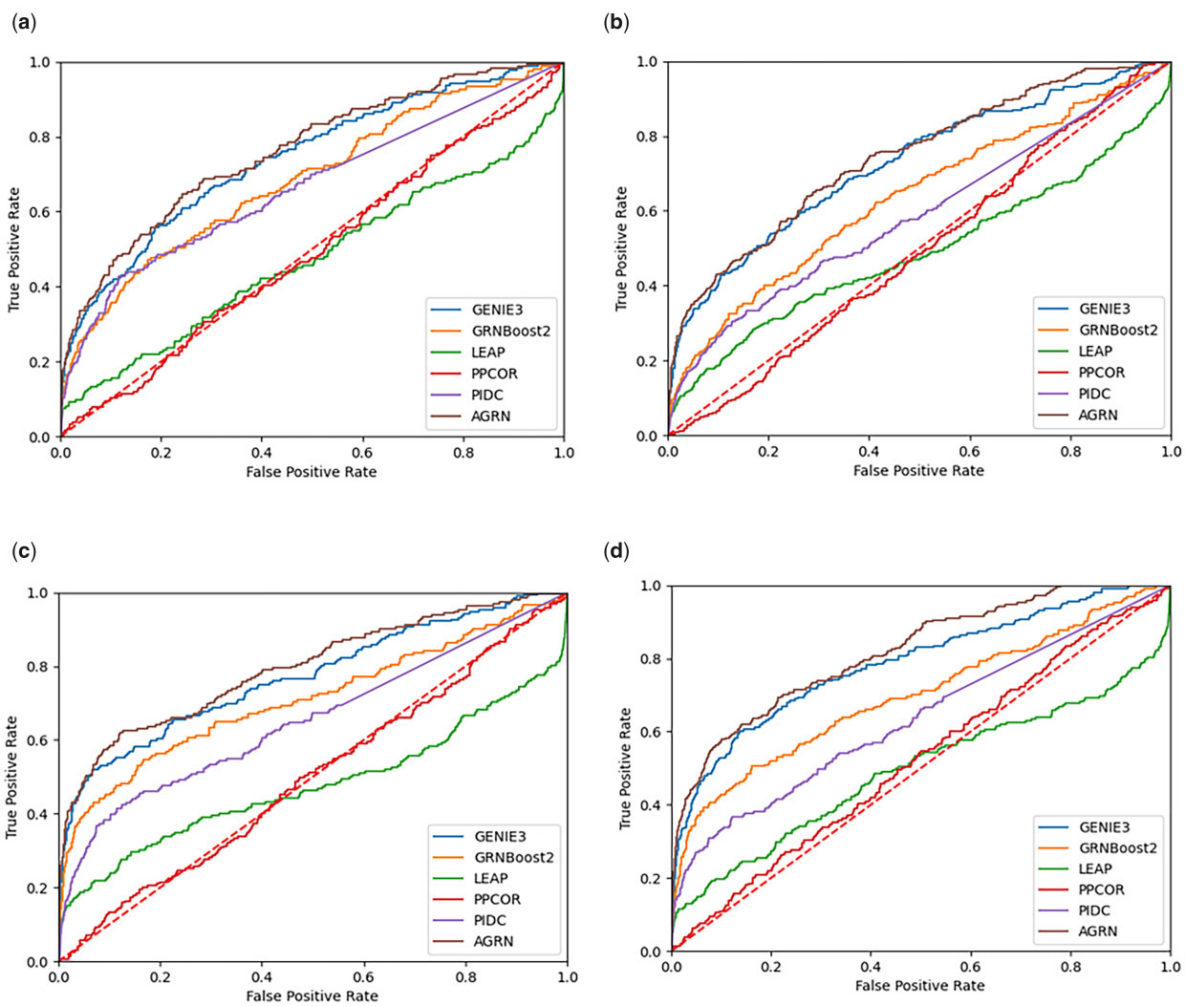
AUROC scores of the compared GRN inference algorithms on four DREAM4 datasets for (**a**) Network1, (**b**) Network2, (**c**) Network3 and (**d**) Network4

**Fig. 3.**
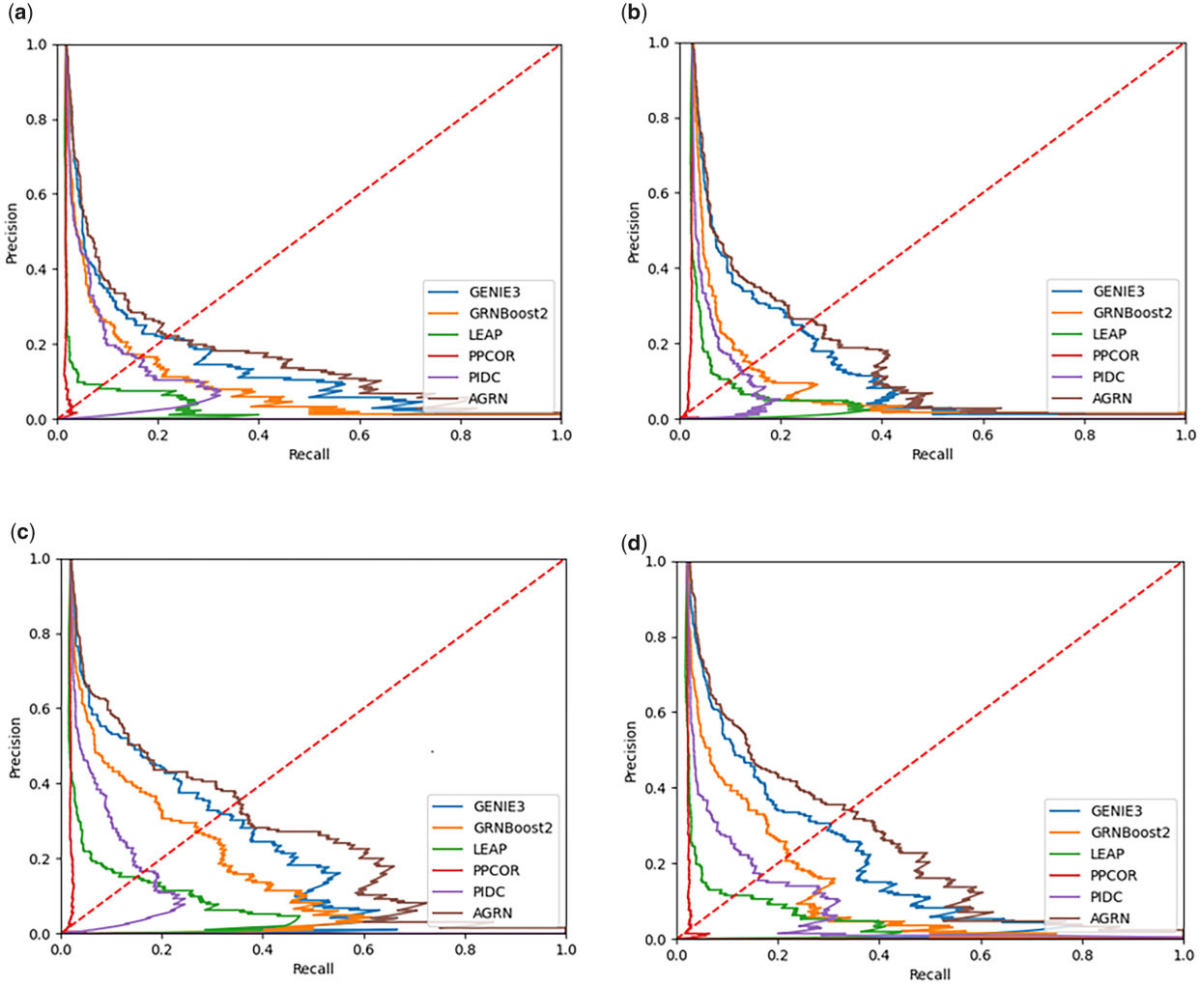
AUPR scores of the compared GRN inference algorithms on four DREAM4 datasets for (**a**) Network1, (**b**) Network2, (**c**) Network3 and (**d**) Network4

Figures 2 and 3 show the AUROC and AUPRC of these compared methods on the four datasets. As can be seen, AGRN outperforms the compared methods on all four simulated datasets from DREAM4. We observed an improvement over the five methods (GRNBOOST2, PPCOR, GENIE3, LEAP and PIDC). Also, AGRN performs better than the widely used method GENIE3.

Compared with the second-ranked method on Network1 in DREAM4, AGRN has a 2.83% increase in AUROC and a 19.21% increase in AUPRC. On Network 2, AGRN achieves an improvement of 2.48% in AUROC and 13.51% in AUPRC over the second-ranked method. For Network 3 in DREAM4, AGRN has a 3.11% higher AUROC and a 25.23% higher AUPRC than the second-ranked method. In addition, compared with the second-ranked algorithm (GENIE3) on Network4 in DREAM4, AGRN has a 3.94% increase in AUROC and a 26.5% increase in AUPRC.

On the other hand, using *in silico* network from the DREAM5 dataset, AGRN shows an improvement of 3.8% and 8.83% compared to the second-ranked method (GRNBoost2) based on AUROC and AUPR, respectively (Supplementary Figs S10 and S11). Moreover, using *E. coli* network from the DREAM5 dataset, compared with GENIE3, AGRN gains an improvement of 7.65% and 7.14% based on AUROC and AUPR, respectively (Supplementary Figs S10 and S11). On the other hand, compared with the first-ranked method (LEAP) on *S. cerevisiae* network from the DREAM5 dataset, AGRN has a -8.42% decrease in AUROC and a -17.21% decrease in AUPRC. Overall, the results clearly indicate that AGRN, an ensemble of techniques such as ShapBasedOnRFR, ShapBasedOnETR and SVR, may be utilized to infer GRNs more accurately than other comparable methods.

To further visualize how many interactions have been correctly predicted in AGRN, we construct the GRN from Network#5 of the DREAM4 dataset. 72 of the top 100 AGRN predictions are accurate, whereas GENIE3 makes 69 such accurate predictions. Incorrect predictions in AGRN and GENIE3 are 28 and 31, respectively (Supplementary Fig. S9a and b). Thus, AGRN predicted three additional interactions compared with GENIE3, and we presented the false positive interactions in the Venn diagram (Supplementary Fig. S9c). In addition, we calculate the z-score of gene importance scores with their target genes (Supplementary Table S5), and we found that all false positive edges are derived from the three highest z-score values of feature importance calculated by AGRN and GENIE3.

### 3.2 Computer runtime

We optimize the runtime by parallelizing the code that can use all the available processors in a system. The studies were conducted on a 64-processor Linux server with 128 GB of RAM. To predict GRNs, all 64 processors were utilized. We compare the runtimes (in minutes) of our ensemble method (AGRN) and its constituent methods (RFR, ETR and SVR) using the DREAM4 datasets (Supplementary Table S2) and the DREAM5 datasets (Supplementary Table S3). We found that SVR took more runtime than the tree-based methods, RFR and ETR. In AGRN, we optimize the hyperparameter of SVR for each target gene which is computationally expensive for a large number of genes. However, we found that SHAP-based importance scores perform better in most cases compared with SVR, as shown in Supplementary Figures S2–S5. So, in the AGRN tool, we keep an option to select either the weighted average of SHAP-based RFR and SHAP-based ETR or the weighted average of all three methods based on the available computing resources. In addition, we compare the runtimes (in minutes) of our ensemble method (AGRN) and the five benchmarking methods (GRNBoost2, PPCOR, PIDC, GEINE3 and LEAP) using three datasets from DREAM5 (Supplementary Table S4). We found that AGRN is the second fastest execution method after PPCOR.

## 4 Conclusion

By combining three disparate machine learning algorithms, we developed an ensemble machine learning method named AGRN that infers GRNs using the importance scores. AGRN achieves competitive performance on both the DREAM4 and DREAM5 datasets. We have analyzed the performance of AGRN using the DREAM4 multifactorial datasets, which include five synthetic networks, and the DREAM5 datasets of synthetic data (*in silico*) and real-world data (*E. coli*). To compare with other algorithms, we used the same settings for the DREAM4 and DREAM5 challenges. In AGRN, we combined the importance scores calculated in each of RFR and ETR based on their Shapley values. In addition, importance scores were calculated from multiple SVR models with iterative sampling. Moreover, we optimize the SVR hyperparameters and use the weighted average of the three methods (ShapBasedOnRFR, ShapBasedOnETR, SVR) to have the final importance scores. The comparison of AGRN with five benchmarking methods (GRNBoost2, PPCOR, GENIE3, LEAP and PIDC) using five networks from the DREAM4 dataset and two datasets from DREAM5 shows that AGRN outperforms the other methods. For example, in Network 1 from DREAM4, AGRN has a 2.83% higher AUROC and a 19.21% higher AUPRC than the second-ranked algorithm. In addition, compared with the second-ranked algorithm on Network4 in DREAM4, AGRN has a 3.94% increase in AUROC and a 26.50% increase in AUPRC. On the other hand, using the *in silico* data from DREAM5, compared with the second-ranked method (GRNBoost2), AGRN achieves an improvement of 2.42% and 8.83% based on AUROC and AUPR scores, respectively. Also, using *E. coli* dataset, the comparison shows that AGRN achieves an improvement of 7.65% and 7.14% based on the AUROC and AUPR scores, respectively. Therefore, these results allow us to conclude that, rather than using a single importance score, AGRN can improve performance on GRN inference by combining importance scores from SHAP-based RFR, SHAP-based ETR and optimized SVR. We believe that the ability of our ensemble method to infer GRN with higher accuracy will have a greater impact on understanding biological systems and disease processes.

The main limitation of this work is the datasets used, DREAM4 and DREAM5, which are from relatively well-studied small model species and contain synthetic data. These are the only benchmark data with experimentally validated gold standard regulatory relationships. With increased regulatory relationship identification, AGRN can be trained and evaluated on more species, including humans, mice and plants.

## Supplementary Material

vbad032_Supplementary_Data
