# Supplementary material for: AGRN: accurate gene regulatory network inference using ensemble machine learning methods
Source: Bioinform Adv. 2023 Apr 5;3(1):vbad032. doi: 10.1093/bioadv/vbad032 (PMC10082608; doi:10.1093/bioadv/vbad032)
Supplement: vbad032_Supplementary_Data [file vbad032_supplementary_data.docx]

AGRN: Accurate Gene Regulatory Network Inference using ensemble machine learning methods

**Duaa Mohammad Alawad^1^, Ataur Katebi^2,3^, Md Wasi Ul Kabir^1^, Md Tamjidul Hoque^1,*^**

**^1^**Department of Computer Science, University of New Orleans, New Orleans, LA, USA.

**^2^**Department of Bioengineering, Northeastern University, Boston, MA, USA.

**^3^**Center for Theoretical Biological Physics, Northeastern University, Boston, MA, USA.

**(**Supplementary Material**)**

Table S1. DREAM4 and DREAM5 Datasets.

| **Dataset** | **No. of Genes** | **No. of TFs** |
| --- | --- | --- |
| DREAM4 Network 1 | 100 | 100 |
| DREAM4 Network 2 | 100 | 100 |
| DREAM4 Network 3 | 100 | 100 |
| DREAM4 Network 4 | 100 | 100 |
| DREAM4 Network 5 | 100 | 100 |
| DREAM5 Network (*In silico*) | 1643 | 195 |
| DREAM5 Network (*E. coli*) | 4511 | 334 |

Table S2. The runtime of AGRN along with RFR, ETR, and SVR using the DREAM4 datasets. The results show the execution time of the parallel (64 cores) (in minutes).

| **Method / Dataset** | **Network 1** | **Network 2** | **Network 3** | **Network 4** | **Network 5** | **Average** |
| --- | --- | --- | --- | --- | --- | --- |
| **ShapBasedOnRFR** | 0.49 | 0.48 | 0.50 | 0.43 | 0.51 | 0.48 |
| **ShapBasedOnETR** | 0.40 | 0.40 | 0.40 | 0.38 | 0.38 | 0.39 |
| **SVR** | 13.83 | 13.74 | 15.14 | 15.36 | 15.91 | 14.80 |
| **AGRN** | 14.72 | 14.61 | 16.04 | 16.16 | 16.80 | 15.67 |

Table S3. The runtime of AGRN along with RFR, ETR, and SVR using the DREAM5 datasets. The results show the execution time of the parallel (64 cores) (in minutes).

| **Method / Dataset** | ***In silico*** | ***E. coli*** |
| --- | --- | --- |
| **ShapBasedOnRFR** | 5.51 | 9.66 |
| **ShapBasedOnETR** | 5.72 | 9.17 |
| **SVR** | 9.40 | 20.32 |
| **AGRN** | 9.64 | 21.15 |

Table S4. The runtime of AGRN, along with five benchmarking methods using the DREAM5 datasets. The results show the execution time of the parallel (64 cores) (in minutes).

| **Method / Dataset** | ***In silico*** | ***E. coli*** | ***S. cerevisiae*** |
| --- | --- | --- | --- |
| **PPCOR** | 1.678 | 9.064 | 19.943 |
| **GENIE3** | 63.51 | 289.31 | 226.41 |
| **PIDC** | 16.5 | 205.22 | 196.26 |
| **GRNBoost2** | 65.03 | 221.26 | 140.29 |
| **LEAP** | 14.54 | 47.45 | 40.69 |
| **AGRN** | 9.58 | 21.01 | 14.56 |

| Table S5. Z-score for feature importance scores that are calculated using GENIE3 and AGRN using Network5 from DREAM4. | |
| --- | --- |
| \| **Source** \| **Target** \| **Z-score** \| \| --- \| --- \| --- \| \| G9 \| G57 \| 7.31 \| \| G85 \| G37 \| 6.50 \| \| G35 \| G43 \| 8.69 \| \| G9 \| G13 \| 6.68 \| \| G60 \| G44 \| 7.17 \| \| G21 \| G37 \| 4.41 \| \| G100 \| G61 \| 9.03 \| \| G85 \| G29 \| 5.87 \| \| G66 \| G7 \| 3.50 \| \| G25 \| G85 \| 3.92 \| \| G25 \| G21 \| 2.99 \| \| G93 \| G77 \| 7.94 \| \| G62 \| G16 \| 6.74 \| \| G86 \| G6 \| 5.96 \| \| G79 \| G6 \| 5.94 \| \| G25 \| G15 \| 4.35 \| \| G62 \| G45 \| 6.22 \| \| G21 \| G85 \| 3.39 \| \| G62 \| G95 \| 8.35 \| \| G24 \| G72 \| 2.62 \| \| G99 \| G50 \| 6.61 \| | \| **Source** \| **Target** \| **Z-score** \| \| --- \| --- \| --- \| \| G21 \| G29 \| 6.74 \| \| G15 \| G29 \| 6.04 \| \| G29 \| G15 \| 5.86 \| \| G53 \| G55 \| 6.22 \| \| G66 \| G7 \| 4.17 \| \| G70 \| G51 \| 2.65 \| \| G99 \| G68 \| 3.08 \| \| G100 \| G74 \| 5.05 \| \| G63 \| G46 \| 5.87 \| \| G15 \| G85 \| 3.32 \| \| G89 \| G37 \| 4.58 \| \| G79 \| G6 \| 7.08 \| \| G38 \| G51 \| 2.30 \| \| G21 \| G85 \| 3.07 \| \| G27 \| G7 \| 3.44 \| \| G21 \| G10 \| 5.43 \| \| G45 \| G74 \| 0.24 \| \| G3 \| G72 \| 0.23 \| |
| 1. Z-score for false positive edges in GENIE3 | 1. Z-score for false positive edges in AGRN |


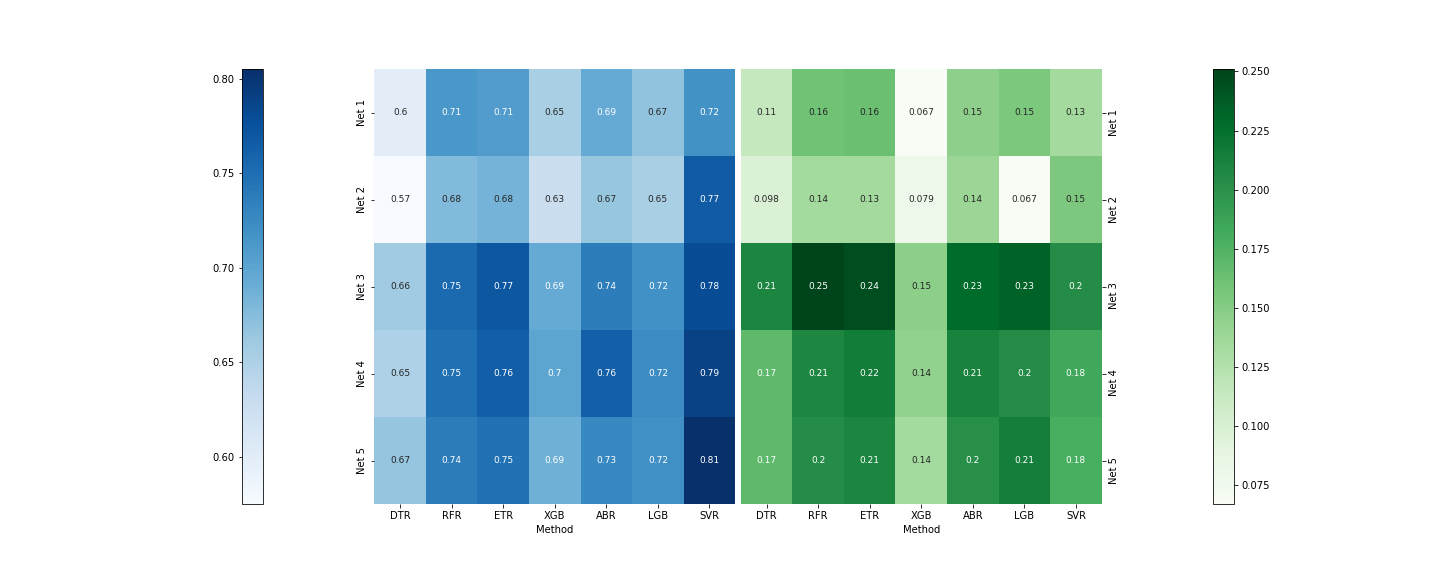


Figure S1: Comparison of individual machine learning methods prediction results in terms of AUROC (left side) and AUPR (right side) on the DREAM4 dataset. (DTR: Decision Tree Regressor, RFR: Random Forest Regressor, ETR: Extra Tree Regressor, XGB: Extreme Gradient Boosting, ABR: AdaBoostRegressor, LGB: Light Gradient Boosted Machine, SVR: Support Vector Regressor)


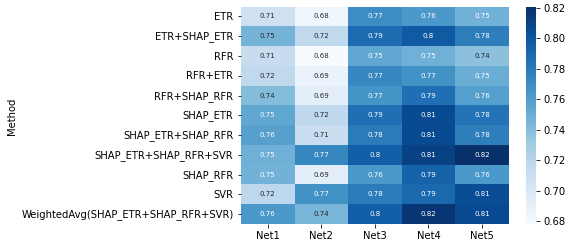


Figure S2: Comparison of different methods in the five Networks dataset of DREAM4 in terms of AUROC metric. (ETR: Extra Tree Regressor, SHAP_ETR: Shapley values as the feature importance scores in Extra Tree Regressor, RFR: Random Forest Regressor, SHAP_RFR: Shapley values as the feature importance scores in Random Forest Regressor, SVR: Support Vector Regressor, WeightedAvg(SHAP_ETR+SHAP_RFR+SVR): weighted average to calculate the aggregate Shapley importance scores in Random forest, Extra Tree Regressor and the feature importance in Support Vector Regressor. '+' indicates rankwise averaging of several methods.


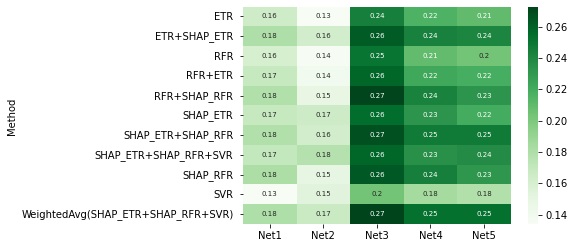


Figure S3: Comparison of different methods in the five Networks dataset of DREAM4 in terms of AUPR metric. (ETR: Extra Tree Regressor, SHAP_ETR: Shapley values as the feature importance scores in Extra Tree Regressor, RFR: Random Forest Regressor, SHAP_RFR: Shapley values as the feature importance scores in Random Forest Regressor, SVR: Support Vector Regressor, WeightedAvg(SHAP_ETR+SHAP_RFR+SVR): weighted average to calculate the aggregate Shapley importance scores in Random Forest, Extra Tree Regressor and the feature importance in Support Vector Regressor. '+' indicates rankwise averaging of several methods.


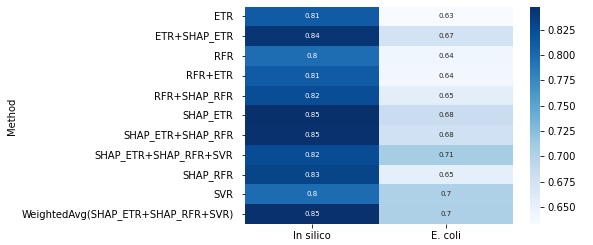


Figure S4: Comparison of different methods in the *In silico and E. coli* Networks dataset of DREAM5 in terms of AUROC metric. (ETR: Extra Tree Regressor, SHAP_ETR: Shapley values as the feature importance scores in Extra Tree Regressor, RFR: Random Forest Regressor, SHAP_RFR: Shapley values as the feature importance scores in Random Forest Regressor, SVR: Support Vector Regressor, WeightedAvg(SHAP_ETR+SHAP_RFR+SVR): weighted average to calculate the aggregate Shapley importance scores in Random forest, Extra Tree Regressor and the feature importance in Support Vector Regressor. '+' indicates rankwise averaging of several methods.


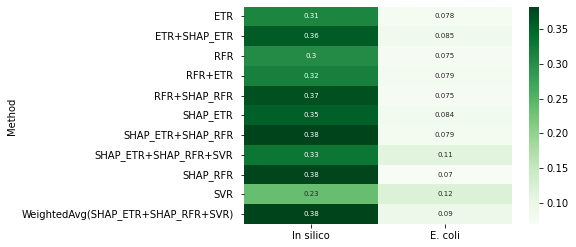


Figure S5: Comparison of different methods in the *In silico and E. coli* Networks dataset of DREAM5 in terms of AUPR metric. (ETR: Extra Tree Regressor, SHAP_ETR: Shapley values as the feature importance scores in Extra Tree Regressor, RFR: Random Forest Regressor, SHAP_RFR: Shapley values as the feature importance scores in Random Forest Regressor, SVR: Support Vector Regressor, WeightedAvg(SHAP_ETR+SHAP_RFR+SVR): weighted average to calculate the aggregate Shapley importance scores in Random forest, Extra Tree Regressor and the feature importance in Support Vector Regressor. '+' indicates rankwise averaging of several methods.

| 1. **DREAM4 Network#1** | 1. **DREAM4 Network#2** |
| --- | --- |
| \| **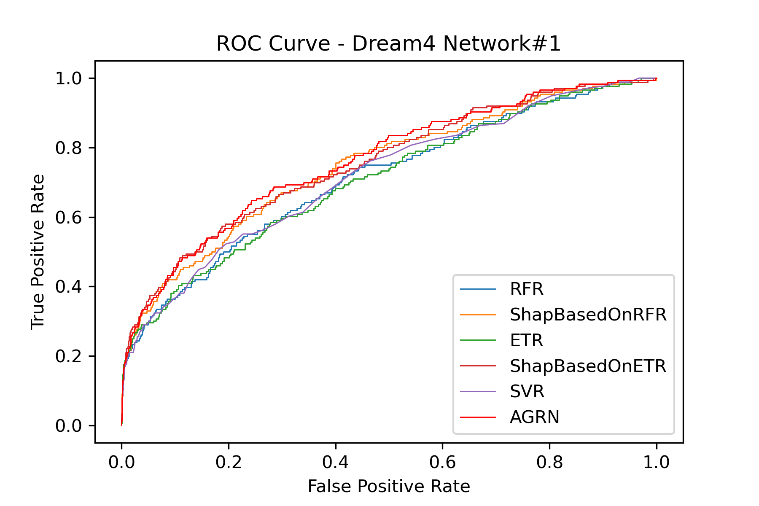** \| \| --- \| | \| **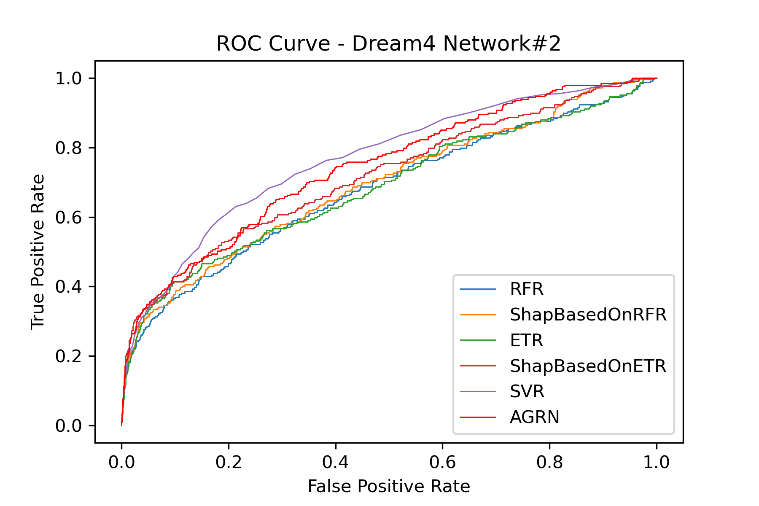** \| \| --- \| |
| 1. **DREAM4 Network#3** | 1. **DREAM4 Network#4** |
| \| **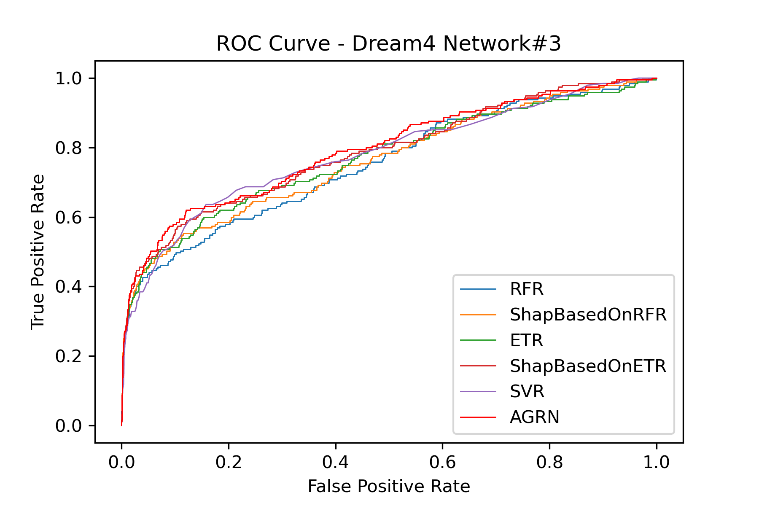** \| \| --- \| | **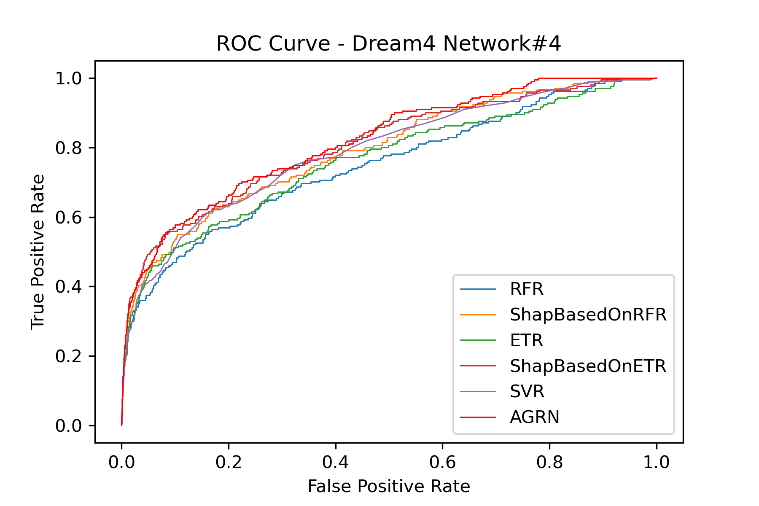** |
| **(e) DREAM4 Network#5** |  |
| **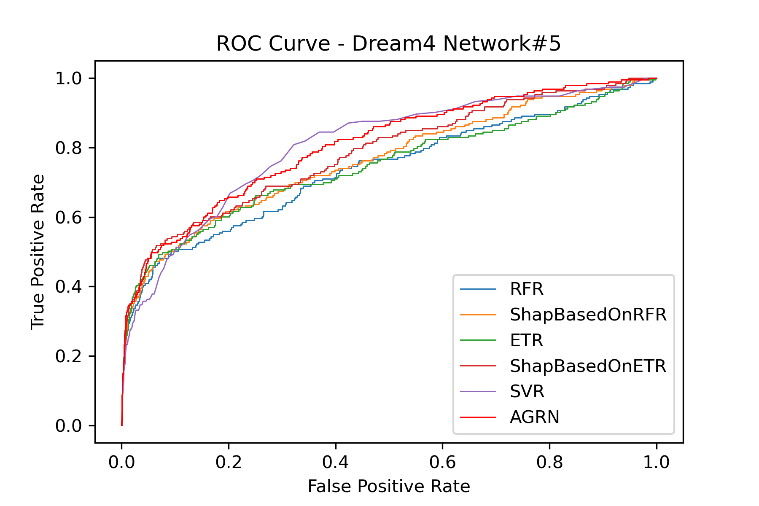** |  |
| **Figure S6:** ROC curve for different methods on the DREAM4 datasets for (a) Network 1, (b) Network 2, (c) Network 3, (d) Network 4, and (e) Network 5. | |

| 1. **DREAM4 Network#1**   **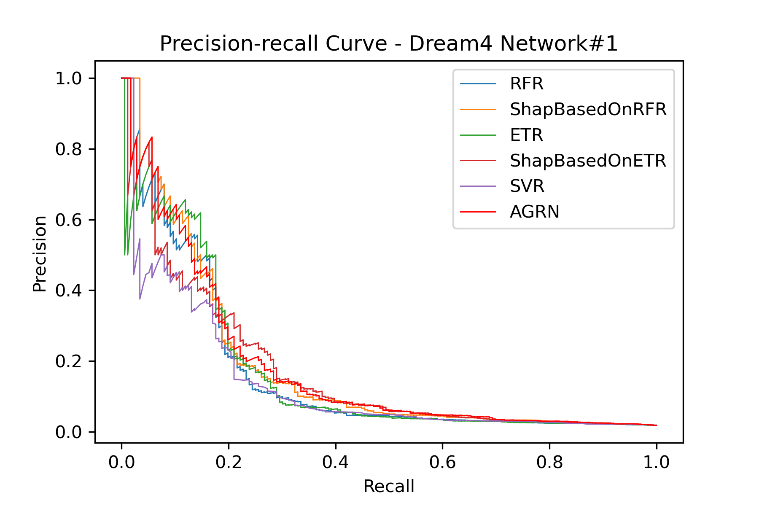** | 1. **DREAM4 Network#2**   **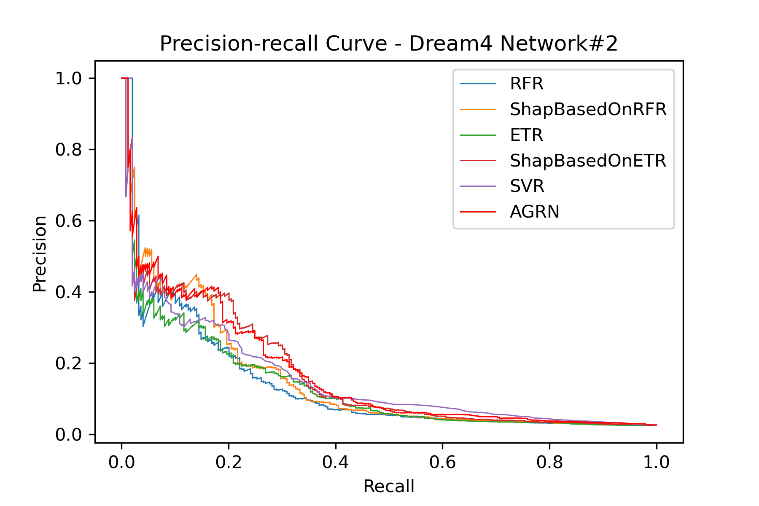** |
| --- | --- |
| 1. **DREAM4 Network#3** | 1. **DREAM4 Network#4** |
| **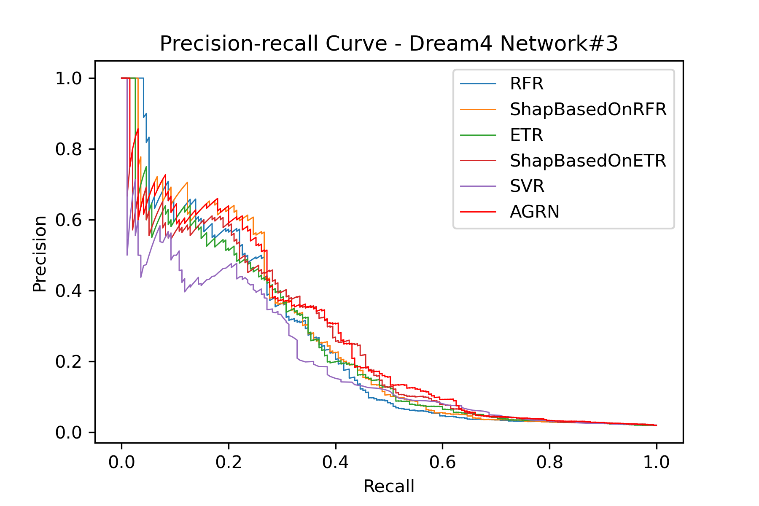** | **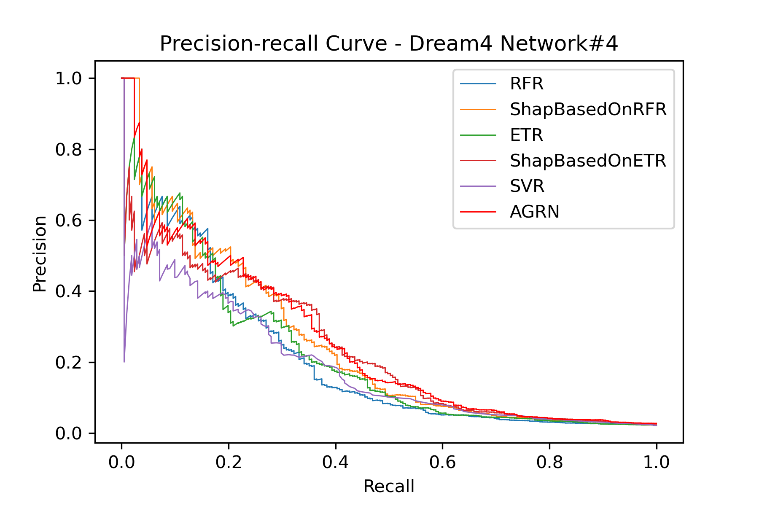** |
| **(e) DREAM4 Network#5** |  |
| **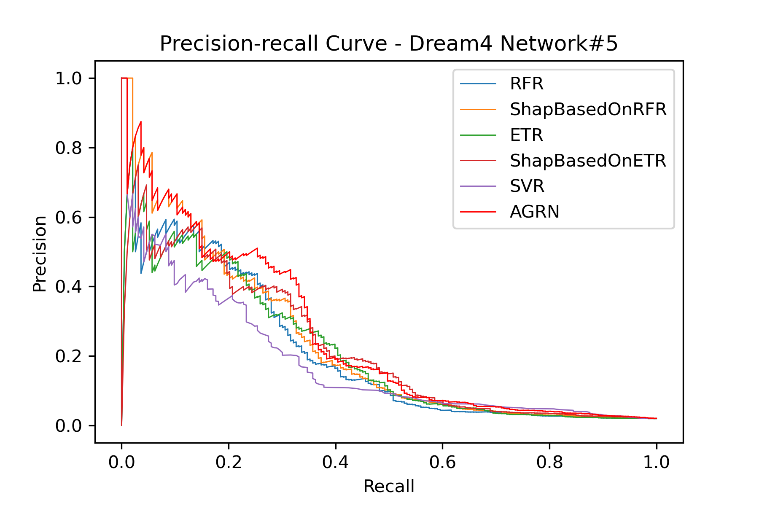** |  |
| **Figure S7:** PR curve for different methods on the DREAM4 datasets for (a) Network 1, (b) Network 2, (c) Network 3, (d) Network 4, and (e) Network 5.  . | |

| 1. **ROC Curve - DREAM5 *in sillico*** | 1. **ROC Curve – DREAM5 *E. coli*** |
| --- | --- |
| **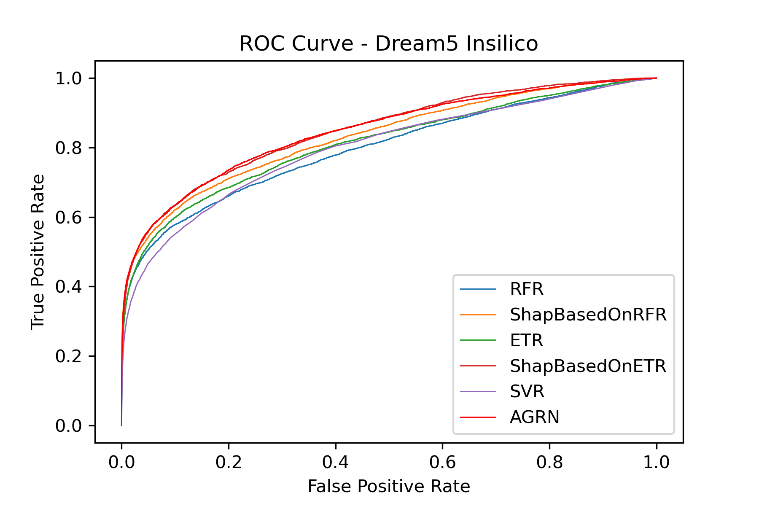** | **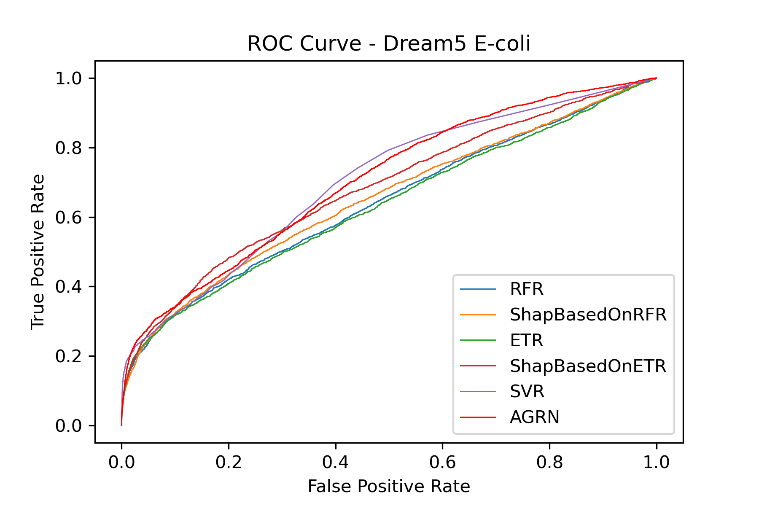** |
| 1. **Precision-Recall Curve – DREAM5 *In silico*** | **(d) Precision-Recall Curve – DREAM5 E. coli** |
| **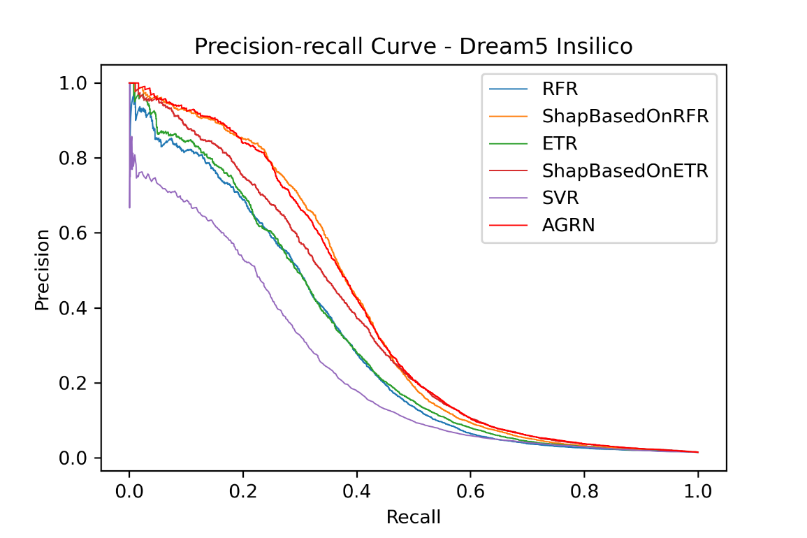** | **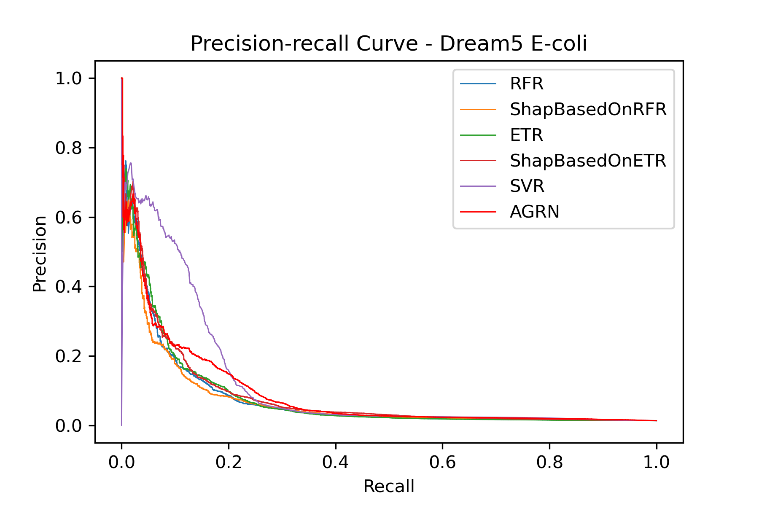** |
| **Figure S8:** ROC curve and Precision-Recall curve for different methods using the DREAM5 (*in silico, E. coli*) dataset. (a) ROC curve for *in silico* data, (b) ROC curve for *E. coli* data, (c) Precision-Recall curve for *in silico* data, and (d) Precision-Recall curve for *E. coli*. | |

| **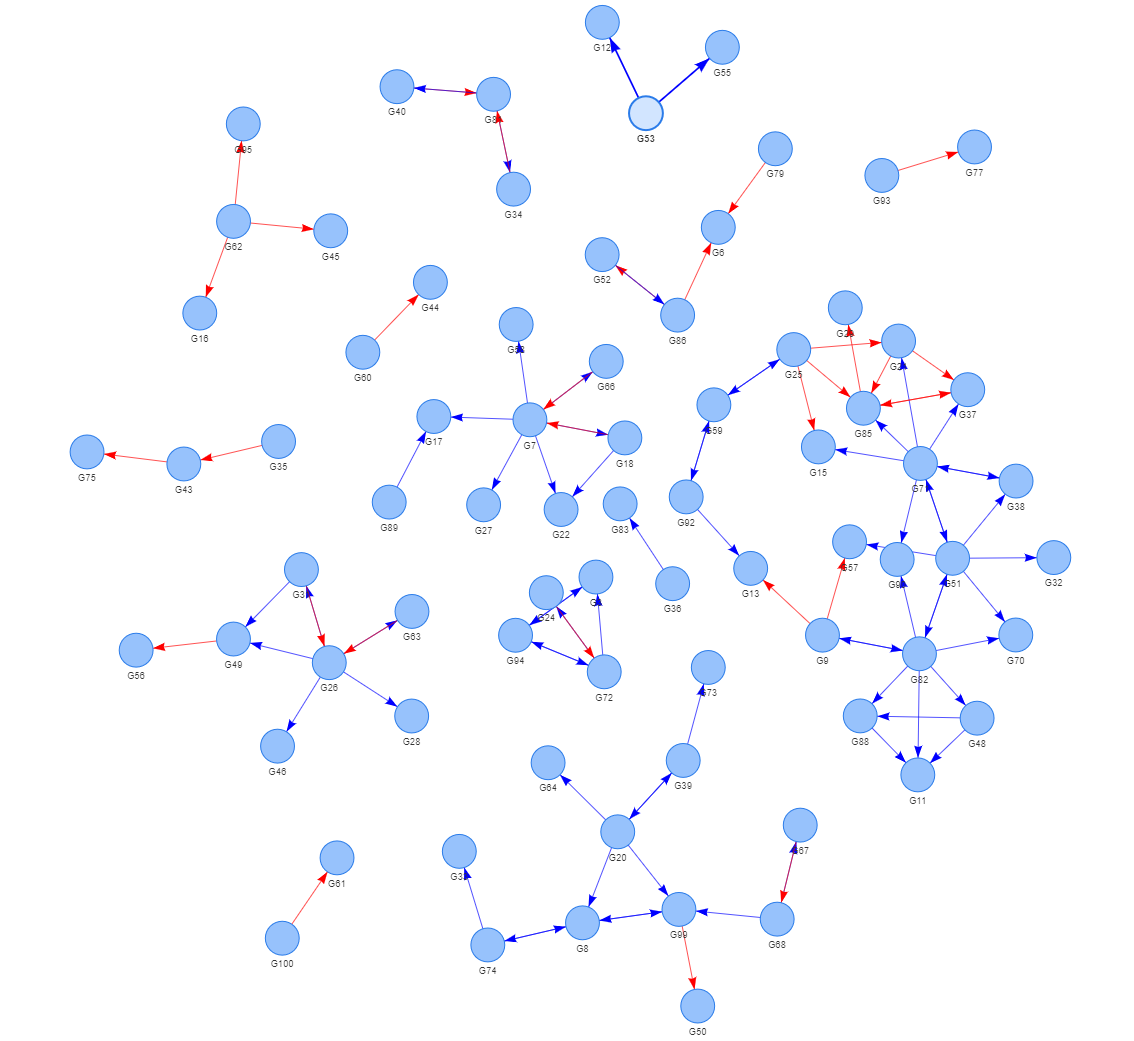** | **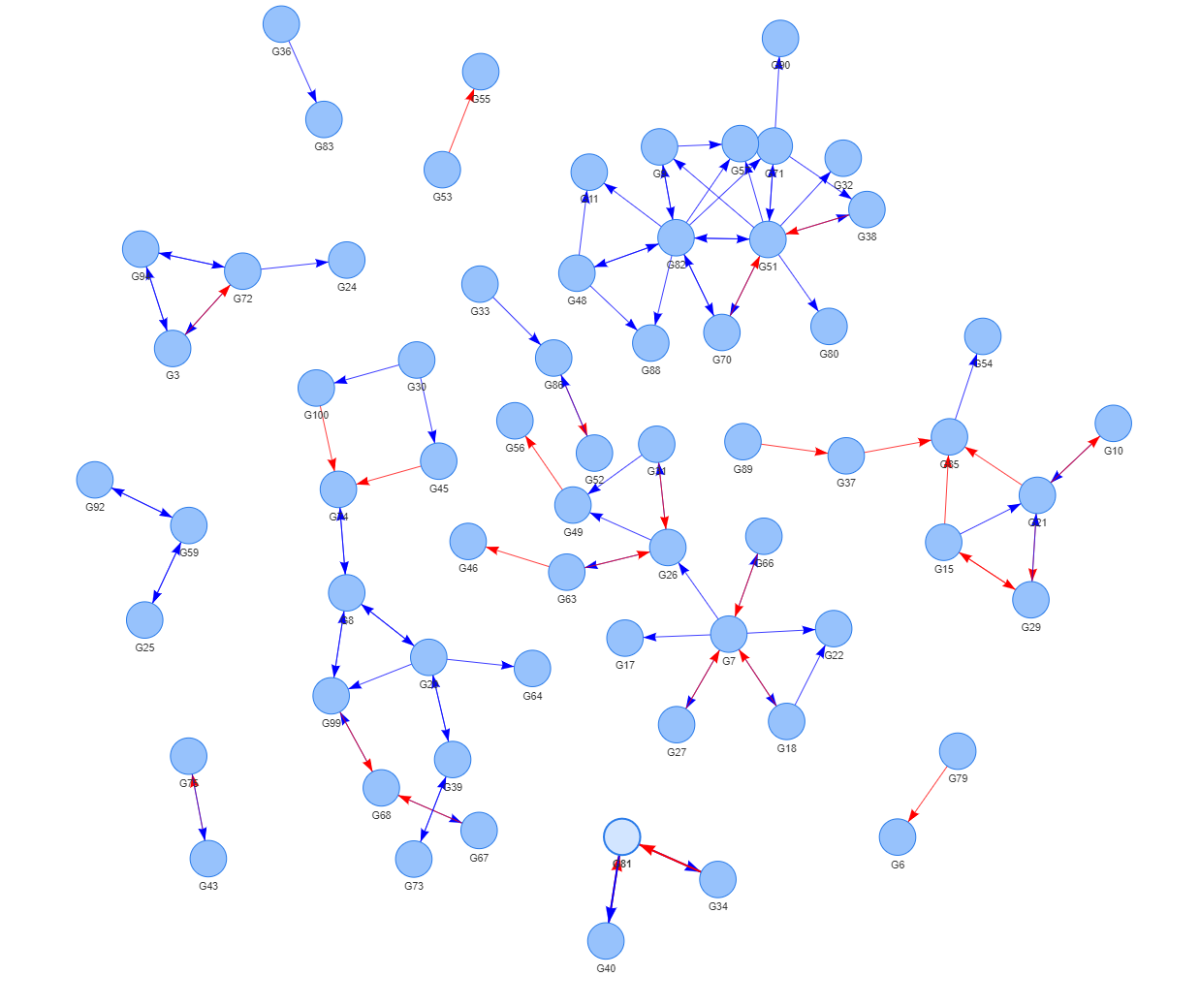** |
| --- | --- |
| 1. **GENIE3 predicted GRN** | 1. **AGRN predicted GRN** |
| **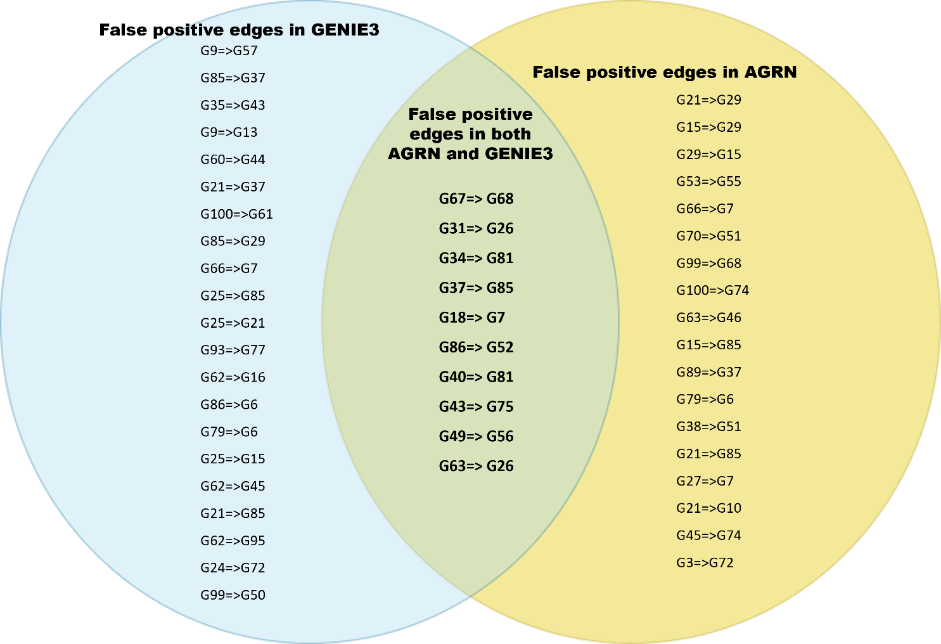**  **(c) Venn diagram representation for False positive edges** | |
| **Figure S9.** Predicted gene regulatory network (GRN) for Network#5 of the DREAM4 dataset. The red edges represent false positives, and the blue edges represent true positives. Python library *pyvis* is used to draw the gene regulatory network. (a) GENIE3 predicted GRN. The number of blue edges is 69, whereas the number of red edges is 31. (b) AGRN predicted GRN. The number of blue edges is 72, whereas the number of red edges is 28. (c) Venn diagram representation of the false positive edges for GENIE3 and AGRN. The yellow circle indicates the false positive edges of AGRN. The blue circle indicates the false positive edges of GENIE3, and the interaction area between the two circles reflects the false positive edges that are shared by both AGRN and GENIE3. | |

|  |  |
| --- | --- |
| **(a ) DREAM5 – *In silico*** | **(b) DREAM5 – *E. coli*** |
| 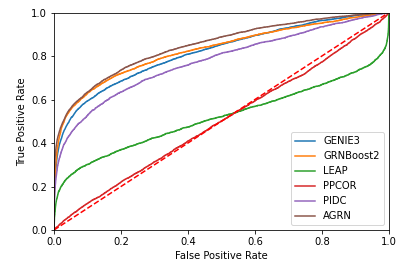 | 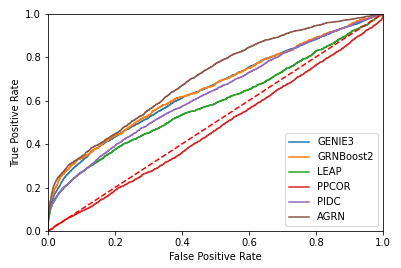 |
| **(c ) DREAM5 –  *S. cerevisiae*** |  |
| 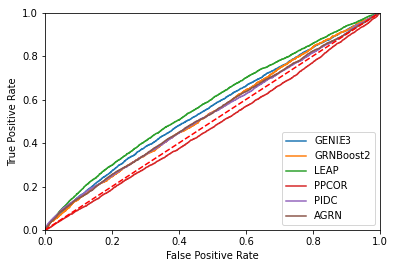 |  |
| **Figure S10**: Comparison of AUROC values of AGRN with other methods using DREAM5 data of (a) *in silico,* (b) *E. coli,* and (c) *S. cerevisiae*. | |

| **(a ) DREAM5 – *In silico*** | **(b ) DREAM5 – *E. coli*** |
| --- | --- |
| 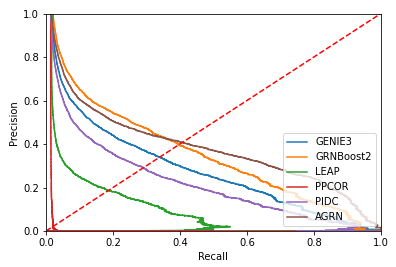 | 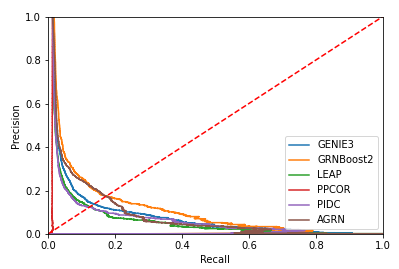 |
| **(c ) DREAM5 –  *S. cerevisiae*** |  |
| 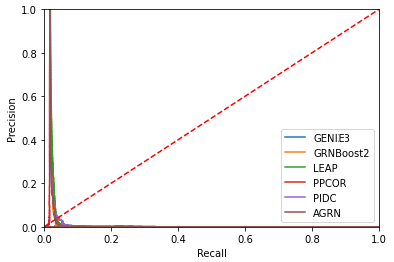 |  |
| **Figure S11**: Comparison of AUPR values of AGRN with other methods using DREAM5 data of (a) *in silico,* (b) *E. coli,* and (c) *S. cerevisiae*. | |
